# ﻿*Vincetoxicumnakaianum* (Asclepiadoideae, Apocynaceae), a new species from Japan for *Cynanchummagnificum* Nakai, nomen nudum

**DOI:** 10.3897/phytokeys.247.125070

**Published:** 2024-10-15

**Authors:** Ko Mochizuki, Shuichi Nemoto, Jin Murata, Tetsuo Ohi-Toma

**Affiliations:** 1 Botanical Gardens, Graduate School of Science, The University of Tokyo, 3-7-1 Hakusan, Bunkyo-ku, Tokyo, 112-0001 Japan The University of Tokyo Tokyo Japan; 2 Faculty of Symbiotic Systems Science, Fukushima University, 1 Kanayagawa, Fukushima, 960-1296 Japan Fukushima University Fukushima Japan; 3 Nature Fieldwork Center, Okayama University of Science, 1-1 Ridai-cho, Kita-ku, Okayama, 700-0005 Japan Okayama University of Science Okayama Japan

**Keywords:** Asclepiadoideae, *Cynanchummagnificum*, Japan, *Vincetoxicummagnificum*

## Abstract

*Vincetoxicum* Wolf is the third largest genus in Asclepiadoideae, and 23 species are distributed in Japan. We discovered that an erect herb species, distributed in the eastern part of the Honshu island, was invalidly named *Vincetoxicummagnificum* (Nakai) Kitag. based on *Cynanchummagnificum* Nakai, nomen nudum. Therefore, we presently name this species *Vincetoxicumnakaianum* K.Mochizuki & Ohi-Toma, and we give a detailed description in this study. Additionally, we provide photographs that demonstrate its ecology and diagnostic characteristics.

## ﻿Introduction

Within the Apocynaceae, the subfamily Asclepiadoideae comprises approximately 3,000 species in 183 genera distributed worldwide (Endress et al. 2018). These plants are characterized by the presence of pollinia, one pollinium per locule, and gynostegia with a highly modified stamen and pistil. The pollinia and stigmata are normally spatially isolated, which suggests that the members of this group strongly rely on animals for pollination (Ollerton and Liede 1997). Because their floral structure physiologically limits access by pollinators, many asclepiads possess specialized pollination systems involving specific animals (Mochizuki et al. 2017; Shuttleworth et al. 2017). The highly specialized pollination systems and the easy assessment of pollinators (determined by the presence of pollinia on the pollinator body) make Asclepiadoideae a good model for studies of pollination (Ollerton and Liede 1997; Ollerton et al. 2019).

*Vincetoxicum* Wolf (tribe Asclepiadeae) is the third largest genus in Ascle­piadoideae, comprising ca. 260 species that geographically extend from the tropics of Africa, Asia, and Oceania to temperate regions from Eurasia (Liede-Schumann et al. 2016; Endress et al. 2018; Liede-Schumann and Meve 2018; POWO 2024). In total, 23 *Vincetoxicum* species are known from Japan, including 16 endemic species (Yamashiro 2017). A recent molecular phylogeny subdivided Japanese *Vincetoxicum* into four groups: a “Far Eastern” clade comprising 11 species endemic to Japan and four wider-ranging species, a single species that was sister to the “Far Eastern clade”, a “subtropical” clade comprising two species, and a “*Vincetoxicum* s. str.” clade comprising five species (Liede-Schumann et al. 2016).

*Vincetoxicummagnificum* (Nakai) Kitag. (Japanese common name: tachi-gashiwa), a perennial herb species endemic to Japan, is closely related to *Vincetoxicummacrophyllum* Siebold. & Zucc. (Japanese common name: tsukushi-gashiwa) and Vincetoxicummacrophyllumvar.nikoense Maxim. (≡ *Cynanchumnikoense* (Maxim.) Makino; Japanese common name: tsuru-gashiwa), which belong to the basal lineage of the “*Vincetoxicum* s.str.” clade (Liede-Schumann et al. 2016). These three taxa have been recognized in several publications thus far, including the recent flora of Japan (Yamazaki 1993; Yamashiro 2017). Recently, the first author introduced *V.magnificum* in Curtis’s Botanical Magazine (Mochizuki et al. 2024); however, we subsequently noticed that the name was not validly published according to the International Code of Nomenclature (ICN, Shenzhen Code; Turland et al. 2018). This species was first published by Nakai (1937) as *Cynanchummagnificum* Nakai, in association with a taxonomic study of related species *V.macrophyllum* (as *Cynanchumgrandifolium* Hemsl.) and V.macrophyllumvar.nikoense (as *Cynanchumnikoense* (Maxim.) Makino). In that publication, the name *Cynanchummagnificum* Nakai was proposed for the “tachi-gashiwa” populations distributed in the Kanto region of Honshu, Japan; however, no Latin description, diagnosis, or even an indirect reference to any former description was provided. Therefore, the name *Cynanchummagnificum* Nakai is a nomen nudum (ICN Art. 38.1 and 39.1). Later, based on this nomen nudum, Kitagawa (1959) published *Vincetoxicummagnificum* (Nakai) Kitag.; therefore, this combination was not validly published (ICN Art. 6.10 and 41.5). In addition, the name cannot be considered as a species novum, “*Vincetoxicummagnificum* Kitag.”, because it was not accompanied by a description, diagnosis, or a reference to a previously and effectively published Latin description or diagnosis (ICN Art. 38.1 and 39.1).

In this study, the Japanese species “tachi-gashiwa” is validly described as *Vincetoxicumnakaianum* K.Mochizuki & Ohi-Toma (ICN Art. 6.9, 38.1, and 39.1), with a detailed description and photographs of living plants. In this case, our proposed name is not “nomen novum” but “species nova” because the names *C.magnificum* and *V.magnificum* have never been validly published (ICN Art. 6.11).

## ﻿Material and methods

To provide a detailed description of *Vincetoxicumnakaianum*, we inspected approximately 50 herbarium specimens in TI for the measurement of vegetative traits, follicles, and seeds. We examined 20 flowers collected from the field (Nikko Botanical Garden, the University of Tokyo) for the measurement of floral traits and dissected 10 flowers to examine staminal traits. The measurement was conducted using ImageJ ver. 1.48 software (Schneider et al. 2012).

We consulted herbarium specimens to establish the distribution information at TI, TUS, KPM, and CBM, as well as digitized specimen images of TNS, ICM, FKSE, BDCJ, and NAC. We examined ca. 160 specimens in total excluding misidentified sheets.

The conservation status of *Vincetoxicumnakaianum* (“tachi-gashiwa”) was calculated following the IUCN Red List categories and criteria v3.1 (IUCN 2012) and IUCN guidelines (IUCN 2019). The Extent of Occurrence (EOO) and Area of Occupancy (AOO) were calculated using the GeoCAT software (Bachman et al. 2011). Further, to complement our calculation, we consulted the red data book published by the Ministry of the Environment of Japan (https://www.env.go.jp/content/900515981.pdf), which is based on the IUCN criterion.

## ﻿Taxonomic treatment

### ﻿ Vincetoxicum nakaianum

Taxon classificationPlantaeGentianalesApocynaceae

K.Mochizuki & Ohi-Toma sp. nov.

1FD55419-6E69-5C40-8AF0-93AE8F009E1D

urn:lsid:ipni.org:names:77350303-1

Figs 1 , 2 , 3

= Cynanchumnikoense (Maxim.) Makino pro parte, Somoku-Dzusetsu, ed. 3. 1: 299 (1907), in nota excl. basionym; Makino, Bot. Mag. (Tokyo) 22: 169 (1908), excl. basionym et synonym; Makino & Nemoto, Fl. Jap.: 329 (1925), quoad descr. tantum; Makino, Ill. Fl. Jap.: 241, f. 460 (1925); Makino, Ill. Fl. Nippon: 207, f. 619 (1940).

#### Type.

Japan, Pref. Tochigi • Nikko city, Nikko Botanical Garden, naturally distributed, 36°44'59.9"N, 139°35'15.1"E, alt. 640 m, 22 May 2021, fl., *K. Mochizuki KMH0484* (holotype: TI[00239769]).

#### Diagnosis.

*Vincetoxicumnakaianum* is morphologically similar to *V.macrophyllum* but is distinguished by an erect stem terminated by an inflorescence, and larger, greenish to brownish flowers, 10–15 mm in diam. (vs. flowers dark purple and 4–5 mm in diam.) with a glabrous corolla (vs. villous).

**Figure 1.**
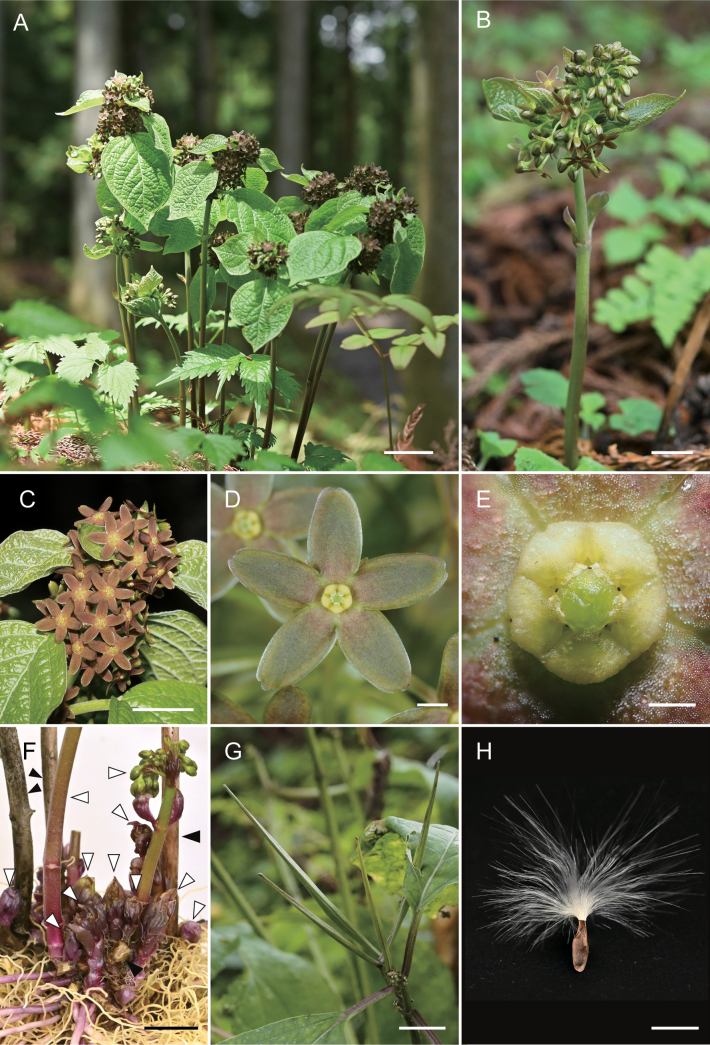
*Vincetoxicumnakaianum* K.Mochizuki & Ohi-Toma **A** habitat and flowering stems of a single individual **B** young inflorescence and coetaneous leaves **C** mature inflorescence **D** flower **E** top view of gynostegium **F** shoots of previous year (black triangles) and current year (white triangles) **G** young follicles **H** seed. Scale bars: 3 cm (**A**); 1 cm (**B, C, F–H**); 2 mm (**D**); 0.5 mm (**E**).

**Figure 2.**
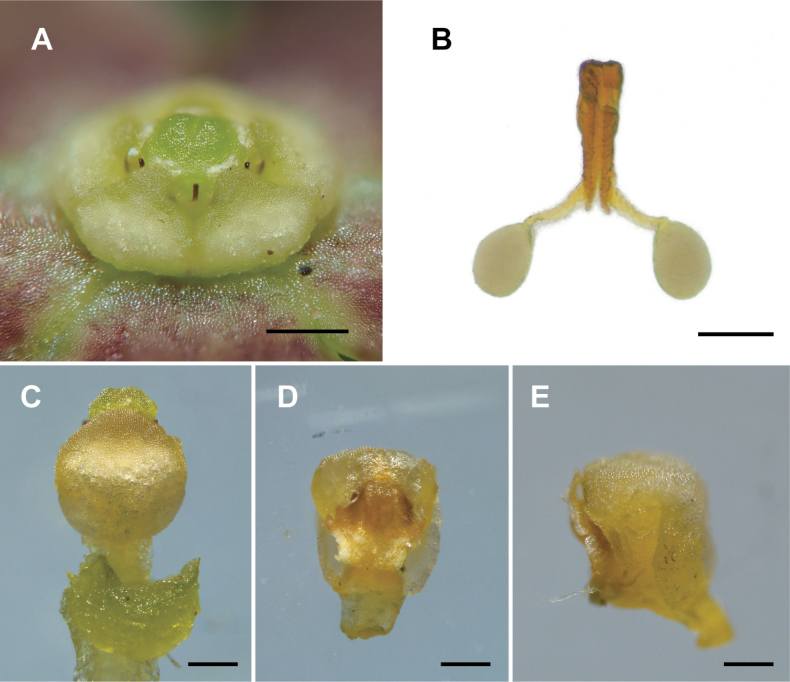
Gynostegium and pollinarium (enlarged) **A** oblique view of gynostegium **B** pollinarium **C** abaxial side of a stamen covered with the corona **D** adaxial side of a stamen **E** side view of a stamen. Scale bars: 0.5 mm (**A, C–E**); 0.1 mm (**B**).

#### Description.

Perennial herb 30–60 cm in height. ***Roots*** fibrous fascicled. ***Rhizome*** short, erect. ***Stems*** erect, weakly pubescent, not branched, 4–6 mm in diam., terminated by an inflorescence. ***Leaves*** opposite, 1–4 pairs crowded near the shoot apex; ***petiole*** 1–4 cm long; ***blade*** broadly ovate to rhombic, 3–8 cm long, 2–6 cm wide when flowering, 8–20 cm long, 5–13 cm wide when fruiting. ***Inflorescence*** terminal and sometimes axillary, densely umbellately cymose; ***flowers*** 10–30, 5-merous, 10–15 mm in diam., weakly pubescent, greenish; ***peduncle*** 5–20 mm long, unbranched; ***pedicels*** glabrous, 5–15 mm long, ca. 0.5 mm in diam., greenish; ***calyx*** rotate, weakly pubescent on abaxial surface, 4–6 mm in diam., deeply lobed, lobes lanceolate, ca. 2 mm long, ca. 0.7 mm wide, apex obtuse to acute; ***corolla*** rotate, 10–15 mm in diam., dull brownish to dull greenish, deeply lobed, lobes oblong, apex obtuse to orbicular, 6–9 mm long, 2–3 mm wide, both surfaces glabrous, apex obtuse to orbicular; ***gynostegial corona*** fleshy, 1.5–2 mm long, 1.5–2 mm in diam., lobes 0.75–1 mm long, ca. 1 mm wide, apex obtuse to orbicular and medially angustate. ***Gynostegium*** 1–1.5 mm in diam., slightly below level of corona; ***style-head*** flattened, 0.5–1 mm in diam. ***Stamen*** 0.6–0.8 mm long, 0.4–0.5 mm wide; anther wings 0.2–0.25 mm long; ***connective appendage*** membranous, ovate, 0.1–0.17 mm long, 0.3–0.4 mm wide. ***Pollinarium***: ***corpusculum*** narrowly oblong, ca. 0.12 mm long; ***caudicles*** oblong, ca. 0.04 mm long, sub-basally connected to corpusculum at a 120° angle; ***pollinium*** ellipsoid, ca. 0.08 mm long, ca. 0.05 mm wide, subapically attached to caudicle. ***Follicles*** usually two per flower, erect, divaricate at a 70°–100° angle, 7–15 cm long, narrow-lanceolate, gradually attenuate to apex. ***Seeds*** brown, slightly winged, narrowly ovate, 10–15 mm long, 2–3.5 mm wide; ***coma*** 2–5 cm long, silver.

**Figure 3.**
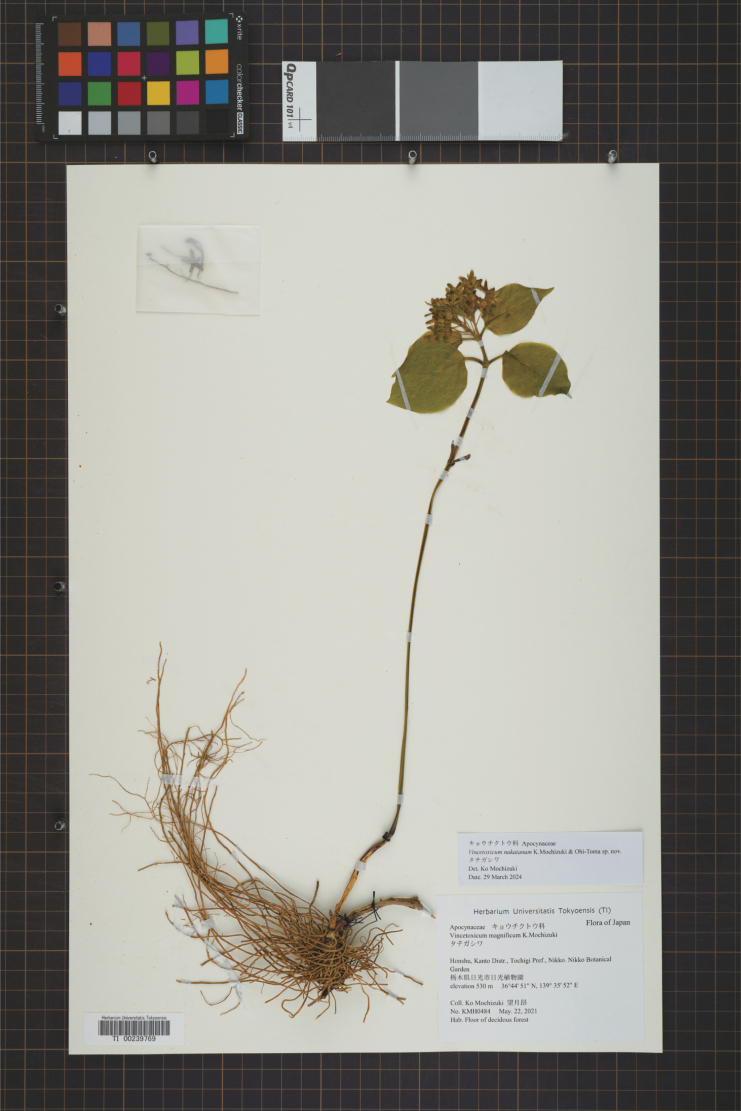
Holotype of *Vincetoxicumnakaianum* K.Mochizuki & Ohi-Toma. (*K.Mochizuki KMH0484*, TI [00239769]).

#### Japanese common name.

Tachi-gashiwa. Makino (1890) was the first to mention the common name “tachi-gashiwa” as a misidentification for *Vincetoxicummacrophyllum* Siebold & Zucc.

#### Phenology.

Flowering from late March to May; fruiting from June to February.

#### Distribution and habitat.

**Figure 4.**
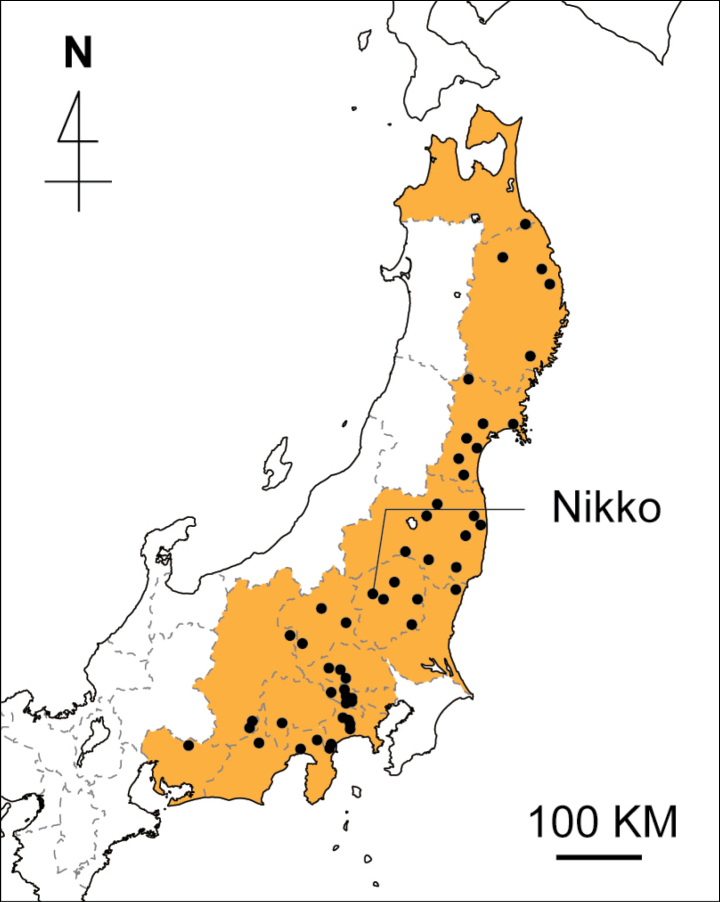
Distribution map of *Vincetoxicumnakaianum* K.Mochizuki & Ohi-Toma. Dots indicate the geographic locations of the specimens examined, including the holotype. Prefectures with records based on herbarium specimens are indicated in orange.

Japan. Alt. ca. 100–850 m. Central to northern Honshu: Prefs. Aomori, Iwate, Miyagi, Fukushima, Gunma, Tochigi, Ibaraki, Tokyo, Kanagawa, Shizuoka, Yamanashi, Nagano, and Aichi (see Additional specimens examined) (Fig. 4). Grows in understories of Japanese ceder plantation forests and deciduous forests dominated by *Fagusjaponica* Maxim., *Abiesfirma* Siebold & Zucc., *Quercus* L., *Acer* L., and *Carpinus* L.

#### Etymology.

The species epithet honors Prof. Takenoshin Nakai (1882–1952), who conferred the name *Cynanchummagnificum* for “tachi-gashiwa”.

#### Conservation status.

Least Concern (LC). *Vincetoxicumnakaianum* is known from many populations throughout the central to northern Honshu island of Japan. Based on the specimen records, the extent of occurrence (EOO) is calculated to be ca. 79.000 km^2^ and the area of occupancy (AOO) is 240 km^2^. However, we should note that this result may be underestimated since the sampling does not cover all the known populations. This species is not listed in the Red Data Book of Japan published by the Ministry of the Environment (Ministry of the Environment 2020). Given that the natural habitat area has not been fragmented or reduced, it is assessed here as Least Concern according to the IUCN criterion (IUCN 2012, 2019).

#### Nomenclatural notes.

The following not validly published “names” correspond with *Vincetoxicumnakaianum* K.Mochizuki & Ohi-Toma.

*Cynanchummagnificum* Nakai, J. Jap. Bot. 8(2): 69 (1937), in textu, nomen nudum. No Latin description, diagnosis, or even an indirect reference to any former description was provided. Therefore, the name *Cynanchummagnificum* Nakai is a nomen nudum (ICN Art. 38.1 and 39.1, Turland et al. 2018)

= *Vincetoxicummagnificum* (Nakai) Kitag., J. Jap. Bot. 34(12): 364 (1959), nomen invalidum.

#### Notes.

*Vincetoxicumnakaianum* is unique in its early flowering habit from late March to May, while the leaves are unfolding, often with the inflorescence blooming before the leaves are fully developed (Fig. 1B). The leaves enlarge from flowering to fruiting, reaching ca. 20 cm long. This flowering habit is rare in the genus *Vincetoxicum*.

Its flowers open in the morning and persist for 2–3 days. The flowers emit a faint odor during daytime, and tiny insects are often observed flying around the inflorescence. Variations in flower color are frequently observed within large populations, ranging from entirely brown to green. In the Nikko area of Tochigi Prefecture on Honshu, this species often co-occurs with V.macrophyllumvar.nikoense; however, their flowering seasons do not overlap, and V.macrophyllumvar.nikoense flowers from June to September.

Although Nakai (1937) cited no specimens, we discovered one specimen (*T. Nakai, s.n.*, TI[00267868]) for which “Cynanchumgrandifolium Hemsley” and “Flores viridescentes” were written on the original label by Nakai; the former was lined out and corrected to “Cynanchummagnificum Nakai, sp. nov.”, also by Nakai.

#### Additional specimens examined.

**Japan**, **Pref. Aomori**: • Hashikami-cho, Mt. Hashikami-dake, 25 Jun. 1946, fr., *J. Koikawa s.n.* (TI[00267869]). **Pref. Iwate**: • Ninohe-gun, Fukuoka-machi, 30 Jun. 1967, wilted fl. and young fr., *H. Hara s.n.* (TI[00267870]) • Iwaizumi-cho, Egawa, 25 Aug. 1973, fr., *M. Takahashi s.n.* (TI[00267871]) • Shimohei-gun, Iwaizumi-machi, 22 Jun. 2000, fr., *K. Yonekura 5634* (TUS[251310]) • Kesen-gun, Sumita-cho, 31 May 2001, fr., *Y. Tazawa s.n.* (TUS[267156]). **Pref. Miyagi**: • Kurihara-shi, Kurikoma, Monzi, Mt. Kurikoma-yama, 24 Jul. 1978, fr., *W. Takahashi 19728* (TUS[465015]) • Kurokawa-gun, Ohmori-yama, 16 May, 1915, fl., *Ogura s.n.* (TI[00267874]) • Akiu, Uenohara, 6 May 1962, fl., *H. Ohashi 2872*, (TI[00267872]) • Oshika-gun, Kinkazan, 23 Jun. 1963, sterile, *M. Takahashi s.n.* (TI[00267873]) • Oshika-gun, Onagawa-cho, Mt. Dairokuten-yama, 5 Aug. 1973, fr., *Y. Sasaki 1409-1* (TUS[321031]) • Iwanuma-shi, Miiroyoshi, 5 Oct. 1993, fr., *T. Mori 8659-c* (TUS[418789]) • Katta-gun, Zao-machi, 18 Jul. 1986, fr., *T. Mori 1607* (TUS[418790]) • Kakuda-shi, Oda, 21 May 1992, fl., *T. Mori 8222-b* (TUS[418791]). **Pref. Fukushima**: • Fukushima-shi, Matsukawamachi-mizuhara, 14 May 2002, fl., *T. Kurosawa 20505* (FKSE[14339]) • Adachi-gun, Ohtama-mura, 8 May 2001, fl., *M. Sato s.n.* (FKSE[94390]) • Sohma-gun, Odaka-machi, 4 May 1969, fl., *N. Sakurai s.n.* (FKSE[33860]) • Futaba-gun, Kashimadaira, 28 Jul. 1962, sterile, *H. Sase, s.n.* (FKSE[6199]) • Tamura-gun, Takine-machi, 30 May 1971, fl., *H. Sase 170-28* (FKSE[6196]) • Iwaki-shi, Tabitomachi, 14 May 2014, fl., *S. Nemoto 1183* (FKSE[82848]) • Ishikawa-gun, Furudono-machi, Okaze, 24 May 2020, fl., *S. Nemoto et al. 5795* (TI[00267875]) • Nishishirakawa-gun, Koseki-mura, date unknown, fl., *N. Imai s.n.* (TNS[31085]). **Pref. Gunma**: • Yamada-gun, Umeda-mura, 10 May 1936, fl., *H. Koidzumi 106893* (TNS[904856]) • Kiryu-shi, Narukamiyama, May 1957, fl., *Y. Asai, s.n.* (TI[00267877]) • Kanra-gun, Shimonita-machi, Kuriyama, 18 Sept. 1954, fr., *T. Wakana WT5856* (CBM[BS5856]) • Kanra-gun, Shimonita-machi, Mt. Arafune-yama, Oct. 1954, sterile., *T. Yamazaki, s.n.* (TI[00270877]) • Kanra-gun, Shimonita-machi, Mt. Myogi-san, 28 May 1954, fl., *K. Sato s.n.* (TI[00270883]). **Pref. Tochigi**: • Nasushiobara-shi, Ooami, 4 Jun. 2005, fl., *M. Tsuchiya 11243* (CBM[BS303509]) • Nikko-shi, Yamakubo, 22 Sept. 2021, fr., *K. Mochizuki KMH0457* (TI[00239771]) • Nasu-gun, Bato-machi, Mt. Torinoko-san, 26 Sept. 1954, fr., *S. Suzuki s.n.* (TUS[243360]) • Kawachi-gun, Kamikawachi-mura, 10 May 1964, fl., *C. Okawa s.n.* (TNS[410216]) • Haga-gun, Mashiko-machi, Mt. Amemaki-yama, 18 May 1952, wilted fl., *S. Suzuki s.n.* (TUS[243359]). **Pref. Ibaraki**: • Kitaibaraki-shi, 3 May 1958, fl., *M. Suzuki s.n.* (TNS[137568]) • Kitaibaraki-shi, Sekimoto-cho, Ogawa, 19 Sept. 2001, sterile, *A. Tamura s.n.* (KPM[0217161]). **Pref. Saitama**: • Iruma-gun, Agano-mura, 17 Oct. 1934, sterlile, *S. Okuyama s.n.* (TNS[663437]) • Chichibu, Buko-san, 24 May, 1953, fl., *S. Kurosawa s.n.* (TI[00267876]) • Hannno-shi, Agano, Mt. Nenoo-gongen, 15 Jun. 1974, sterile, *M. Togashi s.n.* (TI[00271481]). **Pref. Tokyo**: • Mt. Kariyose, 8 May 1930, fl., *T.Nakai s.n.* (TI[00267868]), “Cynanchummagnificum Nakai, sp. nov.,” in sched. • Usui-gun, Usui-machi, 7 Sept. 1952, fr., *I. Yokota 14* (TNS[115037]) • Hachioji-shi, Mt. Jinba, 13 May 1951, fl., *S. Okuyama 9782* (TNS[101552]) • Nishitama-gun, Itsukaichi-machi, 18 Oct. 1970, sterile, *M. Harimoto s.n.* (TNS[754776]) • Ome-shi, May 1955, fl., *T. Satow 8146* (TNS[123870]) • Minamitama-gun, Asakawa-machi, Mt. Takao, 3 May 1921, fl., *T. Ito s.n.* (TNS[396248], TNS[61439]). **Pref. Kanagawa**: • Tsukui-gun, Tsukui-machi, Mt. Yakeyama, 20 Jul. 1980, fl., *S. Kigawa s.n.* (KPM[1000451]) • Aikou-gun, Kiyokawa-mura, 26 Jun. 1981, fr., *H. Takahashi s.n.* (KPM[1000449]) • Minamiashigara-shi, Saijoji, 4 Sept. 1983, fl., *A. Yoshikawa s.n.* (KPM[1000453]) • Isehara-shi, Mt. Ohyama, 2 May 2003, fl., *M. Morikawa s.n.* (KPM[0123462]) • Hadano-shi, Hane, 7 May 2015, fl., *C. Akiko CKD-2-46* (KPM[0182259]) • ibid., 18 Jun. 2015, fr., *C. Akiko CKD-2-166* (KPM[0181449]) • Naka-gun, Isehara-machi, 5 May 1966, fl., *F. Kazami s.n.* (TNS[170266]). **Pref. Shizuoka**: • Gotenba-shi, Higashi-tanaka, 13 Oct. 2021, sterile, *K. Mochizuki KMH0460* (TI[00267878]) • Haibara-gun, Honkawane-cho, Ooma, 19 Jul. 1977, fr., *F. Konta et al. SK301* (TNS[940477]) • Fujinomiya-shi, Fumoto, 30 Aug. 1977, sterile, *F. Konta & T. Masuzawa 2159* (TNS[940473]) • Fujinomiya-shi, Awakura, 8 May 1998, fl., *F. Konta 18501* (TNS[665002]) • Fujinomiya-shi, Saori, 4 May 1985, fl., *T. Sato 7597* (TNS[940476]). **Pref. Yamanashi**: • Minamikomagun, Minobu-cho, 15 Jul. 1951 sterile, *S. Nakagomi* s.n., (BDCJ[2361]). **Pref. Nagano**: • Kitasaku-gun, Karuizawa-machi, 21 Jul. 1936, fr., *K. Shirai s.n.* (TNS[65102]) • Iida-shi, Minamishinano-mura, Tayorigashima, 29 May 2010, fl., *K. Hiruma s.n.* (ICM[HE009442]) • Minamishinano-mura, Tayorigashima, 31 May 2020, *M. Ozeki MOCL200531-10* (NAC[214171]) • Minamishinano-mura, Toyamakawa, 21 Jul. 1936, sterile, *R. Fujiwara 381353-22*, (NAC[65102]). **Pref. Aichi**: • Nishikamo-gun, Obara-mura, Apr. 1954, fl., 16 Oct. 1960, fr., *K. Inami s.n.* (CBM[BS72659], CBM[BS194212]).

## Supplementary Material

XML Treatment for Vincetoxicum nakaianum

## ﻿Additional information

### Author ORCIDs

Ko Mochizuki https://orcid.org/0000-0003-3617-0020

Tetsuo Ohi-Toma https://orcid.org/0000-0002-1936-0220

### Data availability

All of the data that support the findings of this study are available in the main text.
